# Dataset: Repeated and longitudinal measures database from 1994 to 2018 to assess Chile's substance use control policies for the 2000–2010 decade

**DOI:** 10.1016/j.dib.2023.109636

**Published:** 2023-10-02

**Authors:** Karen A. Domínguez-Cancino, Pablo Martínez, José Ignacio Nazif-Munoz

**Affiliations:** a Service sur les dépendances, Faculté de médecine et des sciences de la santé, Université de Sherbrooke – Campus Longueuil, 150, Place Charles-Le Moyne, Bureau 200, Longueuil QC J4K 0A8, Canada; b Center of studies and research in Health and Society (CEISS). University Bernardo O'Higgins, Avenue Viel 1497, 8370993 Santiago, Chile

**Keywords:** Tobacco use, Alcohol use, Drug use, Social determinants of health, Public policy

## Abstract

We developed a database to assess Chile's substance use control policies implemented in the 2000–10 decade. The database includes the measurement of consumption of substances such as alcohol, tobacco, and drugs (cannabis, cocaine, and “*pasta base*” (crack)), individual, relationships, and environmental factors related to substance use, and variables that measure the implementation of laws regulating its use. For the construction of the database, we used information from three sources: i) the biannual National Survey of Drug Consumption for the general population of the National Service of Prevention and Rehabilitation for Drug and alcohol consumption (SENDA) from the Chilean government, ii) the cases filed in local police courts by group of offenses from Chile's Ministry of Justice reports, and iii) the regional imprisoned population from Chile's Correctional Services reports. In the case of the first data source, a data curation process was established to construct this unique database from 1994 to 2018, identifying variables measured systematically over time, standardizing variables' operationalization, and adjusting responses to prespecified flows in each year. On the other hand, substance use control laws enacted in 2004 (alcohol), 2005 (drugs), and 2006 (tobacco) were operationalized as categorical and continuous variables as indicators of its implementation.

Specifications Table

**Table:** 

Subject	Public Health and Health Policy.
Specific subject area	Policy evaluation for substance use control.
Type of data	Table 1: Variables on substance use in Chile from 1994 to 2018 Table 2: Variables on substance control policy in Chile. Table 3: Variables and key information about the standardization process. Table 4: Adjustments considering the flow of the national survey. Table 5: Operationalization of variables on substance control policy.
How the data were acquired	The database considers variables included in the biannual National Survey of Drug Consumption for the general population of the i) National Service of Prevention and Rehabilitation (SENDA) for drug and alcohol consumption in Chile from 1994 to 2018. This organization makes available descriptive reports of each year's results in conjunction with open-access databases in SPSS and STATA formats. We analyzed the reports and databases for each year and created a unique database with relevant variables to the study phenomenon. We also use data available from ii) Reports of Chile's Ministry of Justice and iii) Reports of Chile's Correctional Services. Data from these three sources were merged using a coding prepared in STATA 16.0 and 17.0. Each survey and report included in this database are developed periodically by institutions belonging to the Chilean government. Original surveys and reports are available in Spanish. Translations to English are not available.
Data format	Analyzed
Description of data collection	In the case of the data from the National Survey of Drug Consumption, we identified variables consistently observed over time, standardized the operationalization of the variables using the most common or more straightforward categories available, and cleaned the data considering the flow observed in each survey. For each procedure, we created codebooks in STATA 16.0 or 17.0. For the policy interventions, we identified indicators that reflect the implementation of each law per year.
Data source location	Information was extracted from Chile's Government websites: • Biannual National Survey of Drug Consumption for the general population • Institution: SENDA • City/Town/Region: Santiago, Metropolitan Region. • Country: Chile • Original databases and reports included in the repository. • Correctional services • Institution: Ministry of Justice • City/Town/Region: Santiago, Metropolitan Region. • Country: Chile • Original annual correctional services reports included in the repository • Annual Justice Report • Institution: National Institute of Statistics (INE) • City/Town/Region: Santiago, Metropolitan Region • Country: Chile • Original Annual Justice Report included in the repository
Data accessibility	Repository name: OSFHOME Data identification number: c37mp Direct URL to data: https://osf.io/c37mp/ Dominguez-Cancino, K. A., Nazif-Munoz, J. I., & Martinez, P. (2023, June 8). Repeated and longitudinal measures database from 1994 to 2018 to assess Chile's substance use control policies for the 2000–2010 decade. Retrieved from osf.io/c37mp

## 1 Value of the Data

• The database consolidates the measurement of variables on the consumption (as the prevalence of use during life, last consumption, and current pattern of consumption) of substances such as alcohol, tobacco, and drugs (i.e., cannabis, cocaine, and “*pasta base*” (crack)) and individual, relationships, and environmental factors related to substance use.
• The variables are standardized with biannual measure points from 1994 to 2018, consolidating 24 years of data.
• The database captures the information with its geographical distribution by commune and regions of the country allowing the description and analysis of substance consumption patterns situated in time and place.
• Data points are included before and after the implementation of substance-related control policies in the country.
• Substance-related control policies were operationalized in a simple way to mark the start of the regulations as categorical variables and as continuous variables to assess the possible effects of the policies on consumption.
• The database could be used by both academics and research professionals interested in assessing not only substance-related control policies, including alcohol ones, but also other policies that can indirectly affect substance use outcomes, such as mental health programs in Chile. Relatedly, these data can be reused to specifically address the associations between substance-related control policies, including both national and regional ones, in the outcomes described above, including onset and different frequencies of substance use.

## 2 Objective

This database was generated to assess the association of substance-related control policies that were implemented in the 2000–2010 decade on the consumption of alcohol, tobacco, and drugs in Chile. This approach considers the influence of individual (biological and psychological), socio-cultural, physical environmental factors, and state/government interventions potentially associated with substance use changes over time.

## 3 Data Description

The database is composed of 45 variables. The first 37 variables were created using the data available in the open-access databases of i) the biannual National Survey of Drug Consumption for the general population of SENDA [1]. It includes variables on the consumption as the prevalence of use during life, last consumption, and current pattern of consumption of substances such as alcohol, tobacco, and drugs (i.e., cannabis, cocaine, and “*pasta base*” (crack)) and individual, relationships, and environmental factors related to substance use. The variables extracted from this data source are described in Table 1. We present the name of the variable, the questions used in the survey to obtain the information (authors’ translation), and comments to have a better understanding of the variable included.

**Table 1:** Variables on substance use in Chile from 1994 to 2018.

	Variables	Questions from the survey	Comments
1	year	General information	Year in which the survey was applied
2	Age	General information	Age self-reported by the individual
3	Sex	General information	Sex self-reported by the individual
4	Region	General information	Name of the superior territorial division where the individual resides
5	Commune	General information	Name or number of the basic unit of the local administration of the country
6	Education	What is the last course you passed in regular education?	
7	Marital status	Which is your marital status? What is your current relationship status? (year 1998)	
8	Age-first use tobacco	How old were you when you first smoked cigarettes?	
9	Age-first use alcohol	How old were you when you first consumed alcohol?	
10	Age of first use of cannabis	How old were you when you first consumed this drug?	
11	Age of first use “pasta base”	How old were you when you first consumed this drug?	
12	Age of first use of cocaine	How old were you when you first consumed this drug?	
13	Tobacco use per month	How many cigarettes a day have you smoked in the last 30 days?	
14	Tobacco consumption during life	Have you ever smoked cigarettes in your life?	
15	Alcohol consumption during life	Have you ever drunk alcohol (beer, malt, chicha, wine, champagne) in your life?	
16	Cannabis consumption during life	With the help of these cards, could you tell me which of the following drugs you have consumed at least once in your life by separating them into two piles; those you have consumed and those you have not consumed?	
17	“Pasta base” consumption during life	With the help of these cards, could you tell me which of the following drugs you have consumed at least once in your life by separating them into two piles; those you have consumed and those you have not consumed?	
18	Cocaine consumption during life	With the help of these cards, could you tell me which of the following drugs you have consumed at least once in your life by separating them into two piles; those you have consumed and those you have not consumed?	
19	First tobacco use	When was the first time you smoked cigarettes?	The moment in time of the first use of the substance with delimited categories as options.
20	First alcohol use	When was the first time you consumed alcohol, for example, wine, pisco, beer, chicha, etc.?	The moment in time of the first use of the substance with delimited categories as options.
21	First cannabis use	Was it more than a year ago, less than a year ago, or less than a month ago that you first consumed...	The moment in time of the first use of the substance with delimited categories as options.
22	First “pasta base” use	Was it more than a year ago, less than a year ago, or less than a month ago that you first consumed...	The moment in time of the first use of the substance with delimited categories as options.
23	First cocaine use	Was it more than a year ago, less than a year ago, or less than a month ago that you first consumed...	The moment in time of the first use of the substance with delimited categories as options.
24	Last tobacco use	When was the last time you smoked a cigarette?	The moment in time of the last use of the substance with delimited categories as options.
25	Last alcohol use	When was the last time you consumed alcohol (wine, pisco, beer, chicha, etc.)?	The moment in time of the last use of the substance with delimited categories as options.
26	Last cannabis use	When was the last time you used it?	The moment in time of the last use of the substance with delimited categories as options.
27	Last “pasta base” use	When was the last time you used it?	The moment in time of the last use of the substance with delimited categories as options.
28	Last cocaine use	When was the last time you used it?	The moment in time of the last use of the substance with delimited categories as options.
29	Family drug use	Do any of your current or former family members use or have used drugs?	Variable not measured between 2012 and 2018.
30	Friends drug use	According to what you know: How many of your friends use drugs (available up to 1996). To the best of your knowledge, how many of your friends use drugs (available up to 1998). And as far as you know, do any of your close friends, i.e., those with whom you see frequently, use any of these drugs (available from 2000)?	
31	Neighborhood consumption	According to what you know, the use of one or more of these drugs in the neighborhood where you live is	Refers to the perception of the severity of consumption in the neighborhood. Variable not measured in 1998, and between 2012 and 2018.
32	Facility to obtain cannabis	Tell me if it would be easy, difficult, or impossible for you to get each of these drugs.	
33	Facility to obtain “pasta base”	Tell me if it would be easy, difficult, or impossible for you to get each of these drugs.	
34	Facility to obtain cocaine	Tell me if it would be easy, difficult, or impossible for you to get each of these drugs.	
35	Drug offering	When was the last time you were offered any of these drugs?	
36	Quality of the neighborhood	Quality of the neighborhood	Evaluation of the interviewer that considers the distribution of the streets and quality of the houses in the neighborhood.
37	Expansion factor		Expansion factor created to achieve representativeness of the sample.

The variables related to the enactment of substance-control policies (38 to 45) were managed in two ways. The first one was a “naïve” operationalization that considered the presence or absence of the three laws analyzed. Given that the three laws were in force in 2007, 2008 was determined as the starting date for the measure. Also, because the tobacco law had another update in 2013, we created a categorical variable that could capture the progression of the Tobacco policy.

On the other hand, in the cases of the laws of drugs and tobacco, we could identify in the metrics used to report on the performance of different institutions of the Chilean government, indicators that could measure the effects of the implementation of the laws. Thus, we created the variables “Tobacco Infraction rate”, and “Drug law prisoner rate” using data presented in ii) the annual Ministry of Justice reports [2], and iii) the annual Services Correctional reports [3], respectively. Both variables show mechanisms aimed at regulating the behavior of the population. For each variable, we present the name, and details about the construction of the indicator (Table 2).

**Table 2:** Variables on substance control policy in Chile.

	Variable	Conceptual definition
38	Law	The variable indicates the moment when the three laws were starting point of the alcohol and drugs law. It considers a “naïve” operationalization as a “presence” and “absence” of the law.
39	Tobacco law	The variable indicates the starting point of the tobacco law. It considers a “naïve” operationalization as an “absence,”, “first law,” and “second law.” The first law was enacted in 2006 (Law 20 105), while the second was implemented on 2013 (Law 20 660)
40	Tobacco infraction rate per 100,000 inhabitants	The number of infractions related to tobacco law registered in the Annual Justice Report of the National Institute of Statistics per 100,000 inhabitants (Census data 2017).
41	Tobacco infraction rate per 100,000 inhabitants lagged (1 year)	The number of infractions related to tobacco law with a 1-year lag registered in the Annual Justice Report of the National Institute of Statistics per 100,000 inhabitants (Census data 2017).
42	Drug law prisoner rate per 100,000 inhabitants	The number of prisoners related to drug law registered in The Annual Gendarmerie report per 100,000 inhabitants.
43	Drug law prisoner rate per 100,000 inhabitants lagged (1 year)	The number of prisoners related to drug law with a 1-year lag registered in The Annual Gendarmerie report per 100,000 inhabitants.
44	Drug law prisoner rate per 1000 imprisoned	The number of prisoners related to drug law registered in The Annual Gendarmerie report per 1000 imprisoned.
45	Drug law prisoner rate per 1000 imprisoned lagged (1 year)	The number of prisoners related to drug law with a 1-year lag registered in The Annual Gendarmerie report per 1000 imprisoned.

## 4 Experimental Design, Materials, and Methods

A data curation process of the surveys compiled from 1994 to 2018 on substance use was established to construct this database. This process was composed of four steps: 1) identification of questions that were consistently observed over time using an Excel Spreadsheet that incorporated all the questions for each year (reduction from 499 to 37 questions); 2) identification of possible questions that could contribute to the understanding of the phenomenon "substance use control policy" considering a theoretical background supported in a literature review and causal graphs proposed by the research team; 3) standardization of the operationalization of the variables identified across time using the most common or more straightforward operationalization with a construction a coding book in STATA 16.0 (Table 3); 4) Recodification of variables considering the flow observed in each survey with a construction a coding book in STATA 17.0 [4] (Table 4). Finally, the variables related to the policy interventions were merged using a coding book in STATA 16.0 (Table 5).

**Table 3:** Variables and key information about the standardization process.

Variables	Standard operationalization	Number of observations and missing data	Comments
year	Year of the survey (1994, 1996, 1998, 2000, 2002, 2004, 2006, 2008, 2010, 2012, 2014, 2016, 2018)	271,818 observations and 0 missing data	Created to identify the year of the survey.
Age		271,808 observations and 10 missing data	
Sex	1= Man 2= woman	271,808 observations and 10 missing data	
Region	1= Tarapacá 2= Antofagasta 3= Atacama 4= Coquimbo 5= Valparaíso 6= O'Higgins 7= Maule 8= Bio Bio 9= Araucanía 10= Los Lagos 11= Aysén 12= Magallanes y Antártica 13= Metropolitan region	271,808 observations and 10 missing data	Over time we observed geopolitical changes going from 13 regions to 16. We kept the original 13 regions.
Commune	Coding from the National Institute of Statistics (INE). 109 codes were available, ranging from 1101 to 15,101 with a corresponding commune name plus 72numbersr without the name.	271,808 observations and 10 missing data	Correspond to the basic unit of the administration of the State. The National Institute of Statistics (INE) developed a codification for each commune identified in the country. The last version available is from 2018. We used this coding to standardize the names of the communes. This procedure was applicated from 1998 to 2018. 1994 and 1996 have just numeric values available.
Education	1= Incomplete or complete higher education. 2= High school incomplete or complete 3= No studies, incomplete or complete primary education. 99= Don't know/No answer	220,432 observations and 51,386 missing data	The survey presented a variety of categories to identify the level of education of the individuals. One of the observations is that the maximum education level was increasing over time. The categories were restricted to the most basic ones observed across the years.
Marital status	1= married 2= single 3= divorced, separated, or annulled. 4= widow 5= other 99= Don't know/No answer	240,153 observations and 31,665 missing data	The survey presented various categories to identify the individual's marital status. Recent surveys included information about living with a couple or not. The categories were restricted to the most basic ones observed across the years. In 1998 this variable was not measured. In this case, the information available was about a romantic relationship.
Marital status2	1= Lives with the partner (married or not) 2= Single 3= In a committed relationship 4= Dating someone 99= No answer	31,665 observations and 240,153 missing data	In 1998 the question about marital status was broader with different categories.
Age-first use of tobacco		180,331 observations and 91,487 missing data	
Age-first use of alcohol		217,290 observations and 54,528 missing data	
Age of first use of cannabis		52,277 observations and 219,541 missing data	
Age of first use “pasta base”		6012 observations and 265,806 missing data	
Age of first use of cocaine		10,111 observations and 261,707 missing data	
Tobacco use per month		123,817 observations and 148,001 missing data	
Tobacco consumption during life	1= No 2= Yes 99= don't know/No answer	271,818 observations and 0 missing data	This variable was not available from 1994 to 1998. The information was derived from the question “first tobacco use”.
Alcohol consumption during life	1= No 2= Yes 99= don't know/No answer	271,818 observations and 0 missing data	This variable was not available from 1994 to 1998. The information was derived from the question “first alcohol use”.
Cannabis consumption during life	1= No 2= Yes 99= don't know/No answer	271,818 observations and 0 missing data	
“Pasta base” consumption during life	1= No 2= Yes 99= don't know/No answer	271,818 observations and 0 missing data	
Cocaine consumption during life	1= No 2= Yes 99= don't know/No answer	271,818 observations and 0 missing data	
First tobacco use	0= Never used 1= More than a year ago 2= More than one month but less than one year ago 3= During the last 30 days 99= Don't know/No answer	271,818 observations and 0 missing data	Variability in reported categories. It's unified according to the categories observed consistently over time. Descriptive analyses were performed to accomplish an accurate identification of each category. The category never used was created to identify the non-consumer; this information corresponded to missing data in some years given its consideration as a different variable.
First alcohol use	0= Never used 1= More than a year ago 2= More than one month but less than one year ago 3= During the last 30 days 99= Don't know/No answer	271,818 observations and 0 missing data	Variability in reported categories. It's unified according to the categories observed consistently over time. Descriptive analyses were performed to accomplish an accurate identification of each category. The category never used was created to identify the non-consumer; in some years, this information corresponded to missing data.
First cannabis use	1= More than a year ago 2= More than one month but less than one year ago 3= During the last 30 days 99= Don't know/No answer	271,817 observations and 1 missing data	Variability in reported categories. It's unified according to the categories observed consistently over time. Descriptive analyses were performed to accomplish an accurate identification of each category. The category never used was created to identify the non-consumer; in some years, this information corresponded to missing data.
First “pasta base” use	1= More than a year ago 2= More than one month but less than one year ago 3= During the last 30 days 99= Don't know/No answer	271,818 observations and 0 missing data	Variability in reported categories. It's unified according to the categories observed consistently over time. Descriptive analyses were performed to accomplish an accurate identification of each category. The category never used was created to identify the non-consumer; in some years, this information corresponded to missing data.
First cocaine use	1= More than a year ago 2= More than one month but less than one year ago 3= During the last 30 days 99= Don't know/No answer	271,818 observations and 0 missing data	Variability in reported categories. It's unified according to the categories observed consistently over time. Descriptive analyses were performed to accomplish an accurate identification of each category. The category never used was created to identify the non-consumer; in some years, this information corresponded to missing data.
Last tobacco use	1= More than a year ago 2= More than one month but less than one year ago 3= During the last 30 days 88= Non-applicable 99= Don't know/No answer	271,818 observations and 0 missing data	Variability in reported categories. It's unified according to the categories observed consistently over time. Descriptive analyses were performed to accomplish an accurate identification of each category. The category non-applicable was created to identify the non-consumer, initially missing data for the question.
Last alcohol use	1= More than a year ago 2= More than one month but less than one year ago 3= During the last 30 days 88= Non-applicable 99= Don't know/No answer	271,818 observations and 0 missing data	Variability in reported categories. It's unified according to the categories observed consistently over time. Descriptive analyses were performed to accomplish an accurate identification of each category. The category non-applicable was created to identify the non-consumer, initially missing data for the question.
Last cannabis use	1= More than a year ago 2= More than one month but less than one year ago 3= During the last 30 days 88= Non-applicable 99= Don't know/No answer	271,818 observations and 0 missing data	Variability in reported categories. It's unified according to the categories observed consistently over time. Descriptive analyses were performed to accomplish an accurate identification of each category. The category non-applicable was created to identify the non-consumer, initially missing data for the question.
Last “pasta base” use	1= More than a year ago 2= More than one month but less than one year ago 3= During the last 30 days 88= Non-applicable 99= Don't know/No answer	271,818 observations and 0 missing data	Variability in reported categories. It's unified according to the categories observed consistently over time. Descriptive analyses were performed to accomplish an accurate identification of each category. The category non-applicable was created to identify the non-consumer, initially missing data for the question.
Last cocaine use	1= More than a year ago 2= More than one month but less than one year ago 3= During the last 30 days 88= Non-applicable 99= Don't know/No answer	271,818 observations and 0 missing data	Variability in reported categories. It's unified according to the categories observed consistently over time. Descriptive analyses were performed to accomplish an accurate identification of each category. The category non-applicable was created to identify the non-consumer, initially missing data for the question.
Family drug use	1= No 2= Yes 99= Don't know/No answer	271,818 observations and 0 missing data	From 2000 this question was made for each drug. The answer was unified in one question.
Friends drug use	1= No 2= Yes 99= Don't know/No answer	195,977 observations and 75,841 missing data	From 2000 this question was made for each drug. The answer was unified in one question.
Neighborhood consumption	1= It doesn't exist 2= Mild 3= Serious 99= Don't know/No answer	165,312 observations and 107,506 missing data	
Facility to obtain cannabis	1= Could not get 2= It would be difficult or very difficult 3= It would be very easy or easy 99= Don't know/no answer	271,811 observations and 7 missing data	Variability in reported categories. It's unified according to the categories observed consistently over time.
Facility to obtain “pasta base”	1= Could not get 2= It would be difficult or very difficult 3= It would be very easy or easy 99= Don't know/no answer	271,815 observations and 3 missing data	Variability in reported categories. It's unified according to the categories observed consistently over time.
Facility to obtain cocaine	1= Could not get 2= It would be difficult or very difficult 3= It would be very easy or easy 99= Don't know/no answer	271,817 observations and 1 missing data	Variability in reported categories. It's unified according to the categories observed consistently over time.
Drug offering	0= No 1= Yes 99= Don't know/No answer	271,811 observations and 7 missing data	The question initially requested information about time with the options: never offered, more than a year ago, more than one month but less than one year ago, during the last 30 days and don't know/no answer. From 2000 this question was made for each drug. The answer was unified in one question with dichotomic responses.
Quality of the neighborhood	1=Elegant residential neighborhood 2=Residential neighborhood, still affluent 3=Commercial neighborhoods or narrow streets 4=Working-class neighborhood or populous neighborhood 5=Neighborhood of improvements and shabby pigsties 99=Don't know/no answer	271,618 observations and 220 missing data	Evaluation made by the interviewer considering the quality of the households and streets. In 1998, categories ranging from 1 to 7 were included, from very bad to very good. The categories were normalized as: very bad=5, bad=5, less then regular=4, more than regular=4, regular=3, good=2, very good=1.
Expansion factor		271,736 observations and 82 missing data	It is derived from a complex sampling of housing with three stages (geographic area, housing, and persons inside the housing). The sampling frame is elaborated considering census information.

**Table 4:** Adjustments considering the flow of the survey.

Variables	Performed action	New operationalization	Number of observations and missing data
year		Year of the survey (1994, 1996, 1998, 2000, 2002, 2004, 2006, 2008, 2010, 2012, 2014, 2016, 2018)	271,808 observations 0 missing data
Age	Eliminating individuals without this data		271,808 observations 0 missing data
Sex	Eliminating individuals without this data	1= Man 2= woman	271,808 observations 0 missing data
Region	Eliminating individuals without this data	1= Tarapacá 2= Antofagasta 3= Atacama 4= Coquimbo 5= Valparaíso 6= O'Higgins 7= Maule 8= Bio Bio 9= Araucanía 10= Los Lagos 11= Aysén 12= Magallanes y Antártica 13= Metropolitan region	271,808 observations 0 missing data
Commune	Eliminating individuals without this data	Coding from the National Institute of Statistics (INE). 109 codes ranged from 1101 to 15,101 with a corresponding name of the commune plus 72 numbers without the commune name.	271,808 observations 0 missing data
Education	Assigning a missing value to the category don't know/No answer	1= Incomplete or complete higher education. 2= High school incomplete or complete 3= No studies, incomplete or complete primary education.	219,000 observations 52,808 missing data
Marital status	Assigning a missing value to the category don't know/No answer	1= married 2= single 3= divorced, separated, or annulled. 4= widow 5= other	239,523 observations 32,285 missing data
Marital status2	Assigning a missing value to the category don't know/No answer	1= Lives with the partner (married or not) 2= Single 3= In a committed relationship 4= Dating someone	31,438 observations 240,370 missing data
Age-first use of tobacco	Replace extreme values with missing data (as 888 and 999). Assigning a missing value when the age of first use was higher than the age reported by the individual.		172,371 observations 99,437 missing data
Age-first use of alcohol	Replace extreme values with missing data (as 888 and 999). Assigning a missing value when the age of first use was higher than the age reported by the individual.		210,728 observations 61,080 missing data
Age of first use of cannabis	Replace extreme values with missing data (as 888 and 999). Assigning a missing value when the age of first use was higher than the age reported by the individual.		49,201 observations 222,607 missing data
Age of first use of “pasta base”	Replace extreme values with missing data (as 888 and 999). Assigning a missing value when the age of first use was higher than the age reported by the individual.		5498 observations 266,310 missing data
Age of first use of cocaine	Replace extreme values with missing data (as 888 and 999). Assigning a missing value when the age of first use was higher than the age reported by the individual.		9329 observations 262,479 missing data
Tobacco use per month	Replace extreme values with missing data (above 87). Assigning a missing value to the first use if the person reported as a never user.		103,382 observations 168,426 missing data
Tobacco consumption during life	Assigning a missing value to the category don't know/No answer	1= No 2= Yes	271,472 observations 336 missing data
Alcohol consumption during life	Assigning a missing value to the category don't know/No answer	1= No 2= Yes	271,402 observations 406 missing data
Cannabis consumption during life	Assigning a missing value to the category don't know/No answer	1= No 2= Yes	270,980 observations 828 missing data
“Pasta base” consumption during life	Assigning a missing value to the category don't know/No answer	1= No 2= Yes	270,736 observations 1072 missing data
Cocaine consumption during life	Assigning a missing value to the category don't know/No answer	1= No 2= Yes	270,879 observations 929 missing data
First tobacco use	Assigning a missing value to the first use if the person reported as a never user. Assigning a missing value to the category never used and don't know/no answer	1= More than a year ago 2= More than one month but less than one year ago 3= During the last 30 days	176,345 observations 95,463 missing data
First alcohol use	Assigning a missing value to the first use if the person reported as a never user. Assigning a missing value to the category never used and don't know/no answer	1= More than a year ago 2= More than one month but less than one year ago 3= During the last 30 days	215,523 observations 56,285 missing data
First cannabis use	Assigning a missing value to the first use if the person reported as a never user. Assigning a missing value to the category never used and don't know/no answer	1= More than a year ago 2= More than one month but less than one year ago 3= During the last 30 days	51,840 observations 219,968 missing data
First “pasta base” use	Assigning a missing value to the first use if the person reported as a never user. Assigning a missing value to the category never used and don't know/no answer	1= More than a year ago 2= More than one month but less than one year ago 3= During the last 30 days	5972 observations 265,836 missing data
First cocaine use	Assigning a missing value to the first use if the person reported as a never user. Assigning a missing value to the category never used and don't know/no answer	1= More than a year ago 2= More than one month but less than one year ago 3= During the last 30 days	10,051 observations 261,757 missing data
Last tobacco use	Assigning a missing value to the last use if the person reported as a never user. Assigning a missing value to the category never used and don't know/no answer	1= More than a year ago 2= More than one month but less than one year ago 3= During the last 30 days	177,058 observations 94,750 missing data
Last alcohol use	Assigning a missing value to the last use if the person reported as a never user. Assigning a missing value to the category never used and don't know/no answer	1= More than a year ago 2= More than one month but less than one year ago 3= During the last 30 days	216,327 observations 55,481 missing data
Last cannabis use	Assigning a missing value to the last use if the person reported as a never user. Assigning a missing value to the category never used and don't know/no answer	1= More than a year ago 2= More than one month but less than one year ago 3= During the last 30 days	51,918 observations 219,890 missing data
Last “pasta base” use	Assigning a missing value to the last use if the person reported as a never user. Assigning a missing value to the category never used and don't know/no answer	1= More than a year ago 2= More than one month but less than one year ago 3= During the last 30 days	5961 observations 265,847 missing data
Last cocaine use	Assigning a missing value to the last use if the person reported as a never user. Assigning a missing value to the category never used and don't know/no answer	1= More than a year ago 2= More than one month but less than one year ago 3= During the last 30 days	10,060 observations 261,748 missing data
Family drug use	Assigning a missing value to the category never used and don't know/no answer	1= No 2= Yes	267,632 observations 4176 missing data
Friends drug use	Assigning a missing value to the category never used and don't know/no answer	1= No 2= Yes	191,919 observations 79,889 missing data
Neighborhood consumption	Assigning a missing value to the category never used and don't know/no answer	1= It doesn't exist 2= Mild 3= Serious	142,173 observations 129,635 missing data
Facility to obtain cannabis	Assigning a missing value to the category never used and don't know/no answer	1= Could not get 2= It would be difficult or very difficult 3= It would be very easy or easy	243,194 observations 28,614 missing data
Facility to obtain “pasta base”	Assigning a missing value to the category never used and don't know/no answer	1= Could not get 2= It would be difficult or very difficult 3= It would be very easy or easy	236,748 observations 35,060 missing data
Facility to obtain cocaine	Assigning a missing value to the category never used and don't know/no answer	1= Could not get 2= It would be difficult or very difficult 3= It would be very easy or easy	235,862 observations 35,946 missing data
Drug offering	Assigning a missing value to the category never used and don't know/no answer	1= No 2= Yes	268,828 observations 2980 missing data
Quality of the neighborhood	Assigning a missing value to the category never used and don't know/no answer	1=Elegant residential neighborhood 2=Residential neighborhood, still affluent 3=Commercial neighborhoods or narrow streets 4=Working-class neighborhood or populous neighborhood 5=Neighborhood of improvements and shabby pigsties	271,298 observations 510 missing data
Expansion factor			271,736 observations 72 missing data

**Table 5:** Operationalization of variables on substance control policy.

Variable	Operationalization	Number of observations and missing data
Law	0= absence of laws (1994–2006) 1= presence of laws (2008–2018)	271,808 observations 0 missing data
Tobacco law	0= absence of law (1994–2006) 1= implementation of first law (2008–2012) 2= implementation of second law (2014–2018)	271,808 observations 0 missing data
Tobacco infraction rate per 100,000 inhabitants	Number	82,103 observations 182,705 missing data
Tobacco infraction rate per 100,000 inhabitants lagged (1 year)	Number	108,530 observations 163,278 missing data
Drug law prisoner rate per 100,000 inhabitants	Number	271,808 observations 0 missing data
Drug law prisoner rate per 100,000 inhabitants lagged (1 year)	Number	252,381 observations 19,427 missing data
Drug law prisoner rate per 1000 imprisoned	Number	271,808 observations 0 missing data
Drug law prisoner rate per 1000 imprisoned lagged (1 year)	Number	252,381 observations 19,427 missing data

## Ethics Statement

This work was developed with open-access governmental data from Chile. The surveys are anonymized in the moment that are available in the governmental repository. We did not conduct human or animal studies, nor collect social media data.

Ethical committee approval was not needed.

## CRediT authorship contribution statement

**Karen A. Domínguez-Cancino:** Conceptualization, Methodology, Software, Data curation, Writing – original draft, Writing – review & editing. **Pablo Martínez:** Conceptualization, Methodology, Data curation, Validation, Writing – review & editing. **José Ignacio Nazif-Munoz:** Conceptualization, Data curation, Writing – review & editing, Supervision.

## Data Availability

Database (Reference data) (OSFHOME)
